# On the Rapidity of the Passage of Some Foreign Substances through the Kidneys

**Published:** 1845-09

**Authors:** 


*On the Rapidity of the Passage of some Foreign Substances through the Kidneys.*—The last number of the Medical Gazette contains a continuation of Mr. Erichsen's experiments upon the patient affected with extroversion of the bladder, with the view of determining the rapidity of the passage of several foreign substances through the kidneys.

Vegetable infusions require, according to him, a longer average time for their passage through the system than the ferrocyanate of potash, and, like the latter, the rapidity of their transmission from the stomach to the blood, and their secretion from this by the kidneys, depends in a great measure on the condition of the digestive process at the time they are taken. Thus, an infusion of galls appeared in the urine in 30, 33, and 36 minutes; an infusion of rhubarb in 22 minutes, infusion of madder in 16 minutes, infusion of uva ursi in 35 minutes, and decoction of logwood in 19 minutes.

The next series of experiments were instituted with the view of determining the length of time which intervened between the administration of the salts of the fixed alkalies, and the change in the reaction of the urine from acid to alkaline. The citrates and tartrates of potass and soda alone were used, because the subject of the experiments could not be induced to take either the carbonates of the alkalies or the liquor potassæ in sufficient quantity.

The rapidity with which the urine was rendered alkaline in these experiments varied--in one instance 28 minutes were required, in another 47 minutes, and in two others 34 and 40 minutes. In two instances the urine continued alkaline until the fourth day. The increase in the amount of the urinary secretion after the salts of soda had been taken was remarkable, and well exhibits the diuretic properties of the alkali. After the citrate of potash had been taken, the quantity of urine did not appear to be much augmented, though it was rendered distinctly alkaline.—*Ibid.*

